# Evolutionary dynamics of molecular markers during local adaptation: a case study in *Drosophila subobscura*

**DOI:** 10.1186/1471-2148-8-66

**Published:** 2008-02-26

**Authors:** Pedro Simões, Marta Pascual, Josiane Santos, Michael R Rose, Margarida Matos

**Affiliations:** 1 Universidade de Lisboa, Faculdade de Ciências da Universidade de Lisboa, Centro de Biologia Ambiental, Departamento de Biologia Animal, Campo Grande, 1749-016 Lisboa, Portugal; 2 Department of Genetics, Faculty of Biology, University of Barcelona, 08028 Barcelona, Spain; 3 Department of Ecology and Evolutionary Biology, University of California, Irvine, California 92697-2525, USA

## Abstract

**Background:**

Natural selection and genetic drift are major forces responsible for temporal genetic changes in populations. Furthermore, these evolutionary forces may interact with each other. Here we study the impact of an ongoing adaptive process at the molecular genetic level by analyzing the temporal genetic changes throughout 40 generations of adaptation to a common laboratory environment. Specifically, genetic variability, population differentiation and demographic structure were compared in two replicated groups of *Drosophila subobscura *populations recently sampled from different wild sources.

**Results:**

We found evidence for a decline in genetic variability through time, along with an increase in genetic differentiation between all populations studied. The observed decline in genetic variability was higher during the first 14 generations of laboratory adaptation. The two groups of replicated populations showed overall similarity in variability patterns. Our results also revealed changing demographic structure of the populations during laboratory evolution, with lower effective population sizes in the early phase of the adaptive process. One of the ten microsatellites analyzed showed a clearly distinct temporal pattern of allele frequency change, suggesting the occurrence of positive selection affecting the region around that particular locus.

**Conclusion:**

Genetic drift was responsible for most of the divergence and loss of variability between and within replicates, with most changes occurring during the first generations of laboratory adaptation. We also found evidence suggesting a selective sweep, despite the low number of molecular markers analyzed. Overall, there was a similarity of evolutionary dynamics at the molecular level in our laboratory populations, despite distinct genetic backgrounds and some differences in phenotypic evolution.

## Background

Evolution in a novel environment involves a complex array of processes that produces both genetic and phenotypic changes. The extent of these changes varies as a function of several forces, such as the selective pressures imposed and the magnitude of genetic drift, as well as the genetic background and prior evolutionary history of the populations concerned. Natural selection is an important evolutionary process affecting differentiation between populations. Different selective regimes foster evolutionary divergence, while common novel selective forces are expected to lead to convergence [1]. Nevertheless, there is no certainty about the evolutionary outcome when multiple selectively differentiated populations adapt to the same environment (e.g., [2]).

An important evolutionary factor leading to differences among populations is genetic drift, particularly in populations with low effective size [3]. Moreover, natural selection and drift may interact, leading to disparate evolutionary outcomes among populations sharing a common environment (see [4,5]). Genetic drift can promote the loss of different alleles among distinct isolated populations, potentially affecting the evolutionary response of selected traits that are influenced by such alleles. In addition, directional selection can reduce effective population size, enhancing the impact of genetic drift on genetic variability within populations and differentiation among them (see [6]).

Experimental evolution can help address these issues through the use of controlled selection regimes, controlled population sizes, and replication, both simultaneous and sequential [7]. In particular, the study of the evolution of laboratory populations since their foundation from the wild allows us to study the effects of population of origin, demographic structure, and the absence of gene flow on the process of evolutionary domestication. This experimental paradigm has the additional interest arising from the common pattern of large population sizes in the natural population(s) of origin, leading typically to laboratory populations with high initial genetic variability. All of this makes the study of adaptation to the laboratory well-suited to the analysis of the roles of selection, genetic drift, and their interaction during evolution in a novel environment [8]. In this setting, the evolutionary dynamics of molecular markers during laboratory adaptation offers the possibility of clarifying the impact of an ongoing adaptive event at the molecular genetic level. Few studies have collected such information in an experimental evolution framework (but see [9,10]).

The joint study of evolutionary changes in selectively-important quantitative traits, such as those that define life histories, and highly polymorphic molecular markers, such as microsatellites, allows us to analyse in further detail the effects of natural selection and genetic drift in the genome of evolving populations.

Multilocus screens have been used as a tool to identify regions of the genome that have undergone positive selection (e.g., [11,12]). These tests rely on the assumption that regions subjected to positive selection will deviate from the neutral pattern that is assumed to be present in the remainder of the genome. Microsatellite loci, given their frequent polymorphism, wide distribution, and abundance in eukaryotic genomes, are particularly suited for these screens [12,13]. Although microsatellite markers are often assumed to be neutral (see [14]), they can be affected by selective forces if linkage disequilibrium with a selected locus occurs, an effect known as "hitchhiking" [15]. The spread of a beneficial allele in an adapting population is expected to cause a reduction of variability in the selected locus and its flanking regions [11,16,17] – a "selective sweep". Studying polymorphic microsatellite markers in populations adapting to a new environment should help evaluate their ability to detect loci that deviate from neutral expectations and, at the same time, might reveal regions of the genome implicated in adaptive processes [11].

Here we present a detailed study of the temporal genetic changes in two sets of replicated populations of *Drosophila subobscura *derived from different foundations in the wild (one from Sintra and one from Arrábida, Portugal) as they undergo adaptation to a common laboratory environment, based on molecular markers.

The phenotypic evolution of these populations in the laboratory environment has already been analyzed through evolutionary trajectories for several life history traits, revealing a clear adaptive response, particularly for fecundity-related traits. Nevertheless, these populations also showed differences in their phenotypic evolutionary rates, particularly during an early phase of the laboratory adaptation process [8]. Bearing this in mind, we compared the genetic variability of microsatellites between these populations, searching for possible associations between neutral genetic variability and their adaptive response.

## Results

### AR and TW genetic variability

All AR and TW populations showed high initial genetic variability, as measured at the third generation of laboratory adaptation (see Table 1). The two groups of populations did not statistically differ with respect to either allele number or expected heterozygosity in any of the three generations analyzed by bifactorial mixed ANOVAs (see Table 2). However, significant differences were observed between loci for both allele number and expected heterozygosity in each generation, with microsatellite locus *dsub14 *presenting the lowest mean allele number and the lowest expected heterozygosity in all generations analyzed [see Additional file 1: Genetic Variability of AR and TW populations]. *Post hoc *Scheffé tests on expected heterozygosity also showed significant differences between *dsub14 *and all other loci (data not shown).

**Table 1:** Genetic variability in AR and TW laboratory populations

Regime	Population	Generation	n^a^	n_A_^b^	H_exp_^c^
AR...........	AR_1_	3	29.3	12.6	0.816
	AR_2_	3	28.9	13.2	0.831
	AR_3_	3	29.5	13.7	0.829
	AR_1_	14	29.5	9.9	0.804
	AR_2_	14	29.5	10.2	0.807
	AR_3_	14	28.8	11.0	0.812
	AR_1_	40	29.4	8.9	0.779
	AR_2_	40	29.6	8.1	0.773
	AR_3_	40	29.1	8.9	0.790

TW...........	TW_1_	3	28.5	13.6	0.835
	TW_2_	3	28.8	14.0	0.838
	TW_3_	3	29.4	14.0	0.828
	TW_1_	14	29.2	10.1	0.791
	TW_2_	14	29.9	10.6	0.812
	TW_3_	14	29.5	10.2	0.760
	TW_1_	40	29.3	9.0	0.738
	TW_2_	40	29.8	9.5	0.753
	TW_3_	40	29.9	8.2	0.764

^a ^Mean number of individuals analyzed per locus.

^b ^Mean allele number per locus.

^c ^Expected mean heterozygosity.

**Table 2:** ANOVA differences in allele number and heterozygosity between AR and TW groups

Generation	*Allele Number*		*Heterozygosity*	
	*F*	*p-value*	*F*	*p-value*
3	4.205	0.071	0.885	0.372
14	0.004	0.954	0.801	0.394
40	0.336	0.577	1.458	0.258

Note.-Tests for comparisons between TW and AR were bifactorial mixed ANOVAs with group (AR and TW) and locus as factors.

There was a significant decline in allele number across generations in both groups of populations (AR: *F*_2,18 _= 27.874, *p *< 0.00001; TW: *F*_2,18 _= 20.956, *p *< 0.0001; bifactorial mixed ANOVA). As for expected heterozygosity, TW populations underwent a significant decline across generations (*F*_2,18 _= 4.527, *p *< 0.026), while AR populations did not (*F*_2,18_= 1.748, *p *< 0.203).

Trifactorial mixed ANOVAs were performed to test for differences in the rate of genetic variability decline between *groups *(AR and TW), *periods *(G3-G14 and G14-G40), and *loci *(see Table 3). The arcsine transformation was applied to the expected heterozygosity values (ratios between generations), to meet the assumption of normality. To allow this transformation, heterozygosity ratios higher than 1 were rounded to unity. This happened mostly for microsatellite *dsub14 *due to a temporal increase in heterozygosity in this particular locus [see Additional file 1]. The rate of decline in genetic variability was significantly different between periods, being higher in the first period (generations 3–14) for both mean allele number per locus and expected heterozygosity. Significant differences in the rate of decline of genetic variation were also found among loci. However, this rate of decline did not differ between groups (see Table 3).

**Table 3:** ANOVA differences in the rate of variability decline between groups, periods and loci

	*Allele Number*		*Heterozygosity*	
Factor	*F*	*p-value*	*F*	*p-value*
Group	1.038	0.335	2.392	0.156
Period	14.371	0.004	9.270	0.014
Locus	2.150	0.034	2.741	0.008
Group*Period	1.636	0.233	0.222	0.649
Group*Locus	0.938	0.497	1.657	0.114
Period*Locus	3.239	0.002	1.334	0.233
Group*Period*Locus	1.017	0.434	1.947	0.057

Note.-A trifactorial mixed model was applied with group (AR and TW) and period (G14/G3 and G40/G14) as fixed factors and locus as random factor.

### AR and TW genetic differentiation

AR and TW groups of populations already differed significantly at generation 3 (*F*_*st *groups _= 0.013, CI 95% = 0.008; 0.018). This differentiation increased at generation 14 (*F*_*st *groups _= 0.041, CI 95% = 0.027; 0.055) but then remained constant at generation 40 (*F*_*st *groups _= 0.038, CI 95% = 0.023; 0.056). At the population level, differentiation between AR and TW populations increased significantly through time (*F*_*st *_= 0.015, CI 95% = 0.009; 0.021 at generation 3; *F*_*st *_= 0.071, CI 95% = 0.059; 0.084 at generation 14; *F*_*st *_= 0.106 CI 95% = 0.089; 0.125 at generation 40) [see also Additional file 2: Pairwise *F*_*st *_comparisons between AR and TW populations].

Genetic differentiation within each group of populations (AR and TW) was also analyzed, in each generation and across generations. No genetic differentiation was obtained at generation 3 either between AR populations (*F*_*st *_= 0.004, CI 95% = -0.0008; 0.0082) or between TW populations (*F*_*st *_= 0.001, CI 95% = -0.003; 0.006). On the other hand, all populations within each group were significantly differentiated by generation 14 (*F*_*st *_AR: 0.026, CI 95% = 0.018; 0.034; *F*_*st *_TW: 0.037, CI 95% = 0.025; 0.052) and 40 (*F*_*st *_AR: 0.064, CI 95% = 0.044; 0.082; *F*_*st *_TW: 0.078, CI 95% = 0.049; 0.108). In each generation, genetic differentiation between populations within each group was not significantly different between TW and AR [see also Additional file 3: Pairwise *F*_*st *_comparisons within and across laboratory generations]. In both groups of populations genetic differentiation increased significantly between generation 3 and 14 (AR and TW) (*F*_*st generations *_= 0.005, CI 95% = 0.002; 0.008 for AR; *F*_*st generations *_= 0.018, CI 95% = 0.005; 0.034 for TW) but not between generations 14 and 40 (*F*_*st generations*_= -0.007, CI 95% = -0.0006; -0.0129 for AR; *F*_*st generations*_= -0.006, CI 95% = -0.015; 0.003 for TW).

### AR and TW effective population sizes

Table 4 presents N_e _estimates for both AR and TW populations during the two periods of laboratory adaptation: the first period (generations 3 to 14), the second period (generations 14 to 40) and also during the overall study (generations 3 to 40) using both a pseudo-likelihood approach and the loss of heterozygosity method. N_e _values were estimated excluding microsatellite locus *dsub14 *from the data, given its extremely low diversity and its increase in heterozygosity between generations 3 and 14. Furthermore, the disparity between *dsub14 *and all other microsatellite loci may be due to non-neutrality at this locus, an assumption of all models estimating N_e_. The case of this particular locus will be addressed further below.

**Table 4:** Estimates of effective population size (N_e_) for AR and TW populations

	AR_1_	AR_2_	AR_3_	TW_1_	TW_2_	TW_3_
*Generations 3 to 14*						
N_e _(pseudo-likelihood)	101.52	109.96	227.36	122.86	134.01	115.85
CI (95%)	(74.51–144.62)	(80.41–156.67)	(144.62–419.8)	(88.14–180.43)	(96.61–196.83)	(84.87–165.25)
						
N_e _(Ht/Ho)	116.80	112.78	146.88	69.33	95.13	49.02
N (census)	841.67	800.00	820.83	816.67	895.83	816.67

*Generations 14 to 40*						
N_e _(pseudo-likelihood)	304.42	268.77	395.01	274.10	313.29	389.06
CI (95%)	(209.72–469.60)	(186.20–411.28)	(255.97–677.39)	(209.88–364.82)	(235.63–425.67)	(275.45–573.51)
						
N_e _(Ht/Ho)	282.10	203.60	437.50	160.70	257.50	-
N (census)	927.78	866.67	875.93	963.33	951.85	965.19

*Generations 3 to 40*						
N_e _(pseudo-likelihood)	274.65	165.83	253.08	230.36	263.19	190.98
CI (95%)	(201.40–384.17)	(127.24–218.88)	(186.01–351.29)	(174.43–309.09)	(198.19–357.58)	(143.87–258.34)
						
N_e _(Ht/Ho)	196.87	163.64	271.90	117.71	170.64	170.83
N (census)	897.30	836.49	871.62	924.59	936.49	916.49

Using either method, effective population size estimates for the first period of laboratory adaptation were significantly lower than those obtained for the second period for both TW and AR groups of populations (*t*-tests using as data points the √N_e _estimates of the three replicate populations; *p *< 0.05 for all estimates; see Table 4).

AR populations presented a significantly higher N_e _than TW populations between generations 3 to 14, according to the loss of heterozygosity method (AR N_e _value = 125.67; TW N_e _value = 71.00; *t*-test; *p *= 0.04). Nevertheless, the AR and TW N_e _estimates obtained using the pseudo-likelihood method for this first period did not differ significantly (*t*-tests; *p *> 0.1; see Table 4). In contrast, all effective population sizes estimates between generations 14 and 40 for all AR and TW populations were not significantly different (*t*-tests; *p *> 0.1; see Table 4).

AR and TW effective population sizes were also not significantly different when all 40 generations of laboratory adaptation were considered, regardless of the estimation method used. N_e_/N ratios ranged between 19.8 to 30.6% in AR populations and between 20.8 to 28.1% in TW populations when using the pseudo-likelihood approach. When using the loss of heterozygosity method N_e _/N ratios for the AR populations ranged between 19.6 to 31.2%, and for the TW populations between 12.7 to 18.2% (see Table 4).

### Testing for positive selection during laboratory adaptation

**Figure 1:**
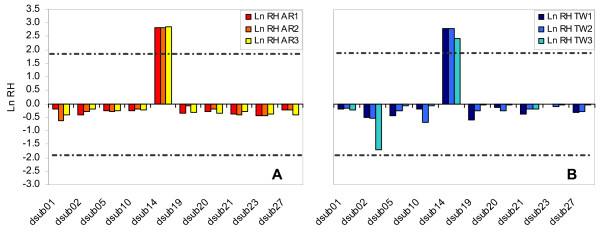
**Standardized Heterozygosity ratios (Ln RH) between generations 3 and 14**. Ln RH ratios (H14/H3) for AR (Fig. 1A) and TW (Fig. 1B) populations. Dashed lines represent the 95% confidence interval of the standardized normal distribution. Positive Ln RH values correspond to increases in variation through time.

Heterozygosity ratios (Ln RH ratios) were calculated for both groups of populations by comparing data between generations 3 and 14 as well as between generations 14 and 40. When comparing generations 3 and 14, Ln RH values were significantly different between loci, both in TW and AR populations (one-way ANOVA; *p *< 0.001). Ln RH values for locus *dsub14 *were significantly different from those obtained for all other loci in all six populations (*post hoc *Scheffé test; *p *< 0.0001 for all comparisons) as a result of the increase in heterozygosity at this locus. Ln RH values between all other pairs of loci were not significantly different (*p *> 0.05 for all comparisons). Also, standardized Ln RH values for microsatellite locus *dsub14 *fell outside the 95% confidence interval of the standard normal distribution for all replicates (see Fig. 1). The pattern observed in locus *dsub14 *was due to the increase in frequency of the same allele (120 bp) in all TW populations and the AR_3 _population, while a different allele (with 116 bp) increased in frequency in both AR_1 _and AR_2 _populations. In TW populations, the allele that increased in frequency (120 bp) rose from an average initial frequency of 11.5% at generation 3 to 31.6% at generation 14. In the AR_3 _population, the 120 bp allele increased from 5% to 19.2% while the 116 bp allele increased in AR_1 _and AR_2 _populations from an average frequency of 5.2% to 15.5%.

**Figure 2:**
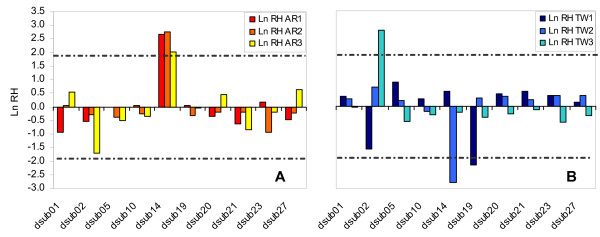
**Standardized Heterozygosity ratios (Ln RH) between generations 14 and 40**. Ln RH ratios (H40/H14) for AR (Fig. 2A) and TW (Fig. 2B) populations. Dashed lines represent the 95% confidence interval of the standardized normal distribution. Positive Ln RH values correspond to increases in variation through time.

Between generations 14 and 40, Ln RH ratios for the AR populations were similar to those mentioned above, again with only locus *dsub14 *significantly differing from all other loci (*post hoc *Scheffé test; *p *< 0.05 for all comparisons). This was also observed for each replicate population by analyzing the standardized Ln RH values (see Fig. 2). During this second period of laboratory evolution, the frequencies of the potentially selected allele in locus *dsub14 *continued to rise in AR_1 _and AR_2 _populations (with the 116 bp allele reaching a frequency of 27.8 and 41.4% at generation 40, respectively). Nevertheless, in the AR_3 _population the allele that had previously increased in frequency (120 bp) slightly decreased (from 19.2% to 13.5%), being the high Ln RH ratio due to the increase in frequency of other alleles. For the TW populations, no significant differences between loci were detected with the general ANOVA or the Scheffé test. However, the analysis of the standardized Ln RH values for each TW replicate population showed some significant results, though they were not consistent among replicates. Specifically, locus *dsub14 *showed a significant decrease in heterozygosity in the TW_2 _population, due to a decrease in frequency of the 120 bp allele. Heterozygosities for this particular locus remained almost constant in the TW_1 _and TW_3 _populations during this period (see Fig. 2).

## Discussion

### Initial microsatellite variability and genetic differentiation

Both AR and TW populations presented similar high levels of initial variability at the ten microsatellite loci studied. Sampling effects of foundation thus apparently did not greatly deplete genetic variability at the start of laboratory culture. The mean allele number (n_A _= 13.4–14.9) and expected heterozygosity (H_exp _= 0.877–0.898) in these populations were similar to the values observed for the same seven common loci studied in other European natural populations (n_A _= 14–16.5, H_exp _= 0.875–0.911, data from [18]).

Nevertheless, AR and TW populations showed significant initial genetic differentiation. The overall *F*_*st *_value of 0.015 at generation 3 was slightly higher than the values obtained from other comparisons among European populations of *Drosophila subobscura *(average *F*_*st *_= 0.006; see [18]). This suggests independent evolution of the ancestral natural populations at the locations from which these populations were derived, Arrábida and Sintra, both in Portugal. These results are somewhat surprising, given the close proximity of the two natural sites where the founders were collected, with a distance of around 50 km. It is possible that the foundation process and the subsequent three generations in the laboratory environment may have accentuated the differences in allele frequencies between these populations. Further sampling and also the analyses of founder individuals may help to clarify whether these two natural populations present restricted gene flow or if their genetic differentiation was just an artefact of laboratory foundation.

### Temporal dynamics of microsatellite variability and genetic differentiation

During the course of 40 generations of laboratory culture, the initially high genetic variability was progressively eroded: both allele number and heterozygosity showed signs of decline during this period. This was predictable, because two forces that are both expected to erode genetic variability – random genetic drift and sustained directional selection – are likely to be pronounced in laboratory cultures, particularly given that effective population sizes are likely to be much lower than those occurring in natural populations of *Drosophila*. This depletion in genetic variability was generally observed throughout laboratory culture for both AR and TW populations, as shown by the parallel declines among variability indexes. However, it is important to note that this loss of genetic variability was relatively mild, since after 40 generations of laboratory adaptation AR and TW populations retained, respectively, 95% and 90% of their initial genetic diversity. The careful maintenance and overall high census sizes (around 900 individuals) in our populations may explain these results. This is in accordance with the high levels of genetic variability that we had already found for our NW *Drosophila subobscura *populations after 49 generations in the laboratory, with 87 to 89% of the genetic diversity of the third generation of TW populations (Simões et al. unpublished data: Divergent evolution of molecular markers during laboratory domestication in *Drosophila subobscura*).

In a recent experimental evolution study with *Drosophila melanogaster *[10], a significant decline in heterozygosity was found, with an estimated loss of 16% for genetic variability in experimental populations maintained with an imposed census size (N≈N_e_) of 100 individuals during 38 generations of laboratory culture after sampling from the wild. The relatively modest decline in genetic diversity observed in this study (5–10%), with a more variable size across generations, suggests the absence of important bottlenecks events during the evolution of our laboratory populations.

The two variability measures used in this study showed similar patterns of decline. Both mean allele number and expected heterozygosity showed a non-linear pattern, with a higher rate of decline in genetic variability between generations 3 and 14. This decline might be the result of a high initial loss of rare alleles due to a bottleneck effect associated with the first generations after foundation from the wild. Such bottleneck effects are expected to lead to a large drop in allele number, though they are not expected to have a major impact on the rate of decline of mean heterozygosity (see [19,20]). The slowing down of the rate of heterozygosity decline through time may instead be a result of a smaller effective population size during the initial generations of laboratory adaptation (see next section).

As a consequence of differential allele loss and allele frequency changes in each population, genetic differentiation (as measured by *F*_*st *_values) increased among all six populations through time. This is expected to be particularly important in smaller populations, due to genetic drift [6]. This led to a progressive divergence among replicate populations within each group, despite their initial lack of differentiation. This genetic differentiation was faster than the differentiation that occurred between the two groups. In fact, in spite of the significant initial differentiation between AR and TW groups the genetic differentiation between populations within each group (*F*_st _TW = 0.078; *F*_st _AR = 0.064) was higher than between groups (*F*_st groups _= 0.038) by generation 40. This suggests that although there were differences in the initial genetic background between the Arrábida and Sintra foundations, they do not seem to have played a central role in the temporal divergence observed among our laboratory populations.

Our results indicate general similarity in the evolutionary dynamics of microsatellite loci during laboratory adaptation across populations. We found no association between the initial genetic variability in molecular markers – which was similar in both groups of populations – and the subsequent phenotypic evolutionary response to the laboratory environment – with a higher adaptive rate for TW relative to AR populations, particularly in the first 14 generations [see 8]. Furthermore, the depletion of genetic variability through time showed only a weak association with the phenotypic evolution of our populations. There was only a suggestion of a higher rate of depletion of heterozygosity in TW populations, which were in fact the ones that presented a higher adaptive rate [8]. Overall, the data suggest that phenotypic adaptation within our laboratory populations had little correlation with the variability shown by molecular markers. Our study suggests caution when inferring adaptive potential from microsatellite data (see also [21-23]; but see [24]) although other laboratory studies covering a wider range of environments and populations are necessary to address this issue.

### Effective population sizes during laboratory adaptation

We found evidence of an increase in the effective size of our laboratory populations through time. The higher selective pressures suffered shortly after laboratory foundation could account for the initial lower effective sizes, since family contributions may vary greatly under strong selection in an initial phase of adaptation [6]. It is however possible that a smaller N_e _in the first period was in part due to an underestimation of the effective size of populations that have just been brought into the lab from the wild, having thus suffered a recent bottleneck. The changes in N_e _across generations may thus reflect allele frequency changes as the populations approach an equilibrium situation. Nevertheless, several simulations done on likelihood based estimation reveal that a severe reduction in population size followed by expansion does not lead to a considerable underestimation of N_e _– less than 5% for an N_e _> 50, as is our case [25]. Moreover, the pseudo-likelihood method of N_e _estimation that we employed is considered relatively robust to frequency changes in rare alleles [26], frequency changes that in our populations occur particularly during the first generations after foundation. Thus, it seems unlikely that the changes in effective population size found using this method – with a three-fold increase between the two periods studied – were chiefly due to the effects of genetic drift in the first generations leading to the loss of rare alleles.

Average N_e _values for the TW populations were systematically lower than those obtained for AR populations. In particular, TW populations presented a significantly lower effective population size when it was estimated using the loss of heterozygosity method applied to the first 14 generations. These results are consistent with the finding of both higher selective pressure (associated with a higher adaptive rate – see [8]) and more genetic drift effects in TW populations (see above).

The N_e_/N ratios obtained in this study – 0.26 for AR and 0.25 for TW populations, according to the pseudo-likelihood estimates – are higher than most estimates based on laboratory-maintained populations. For instance, N_e_/N values below 0.051 were found for captive populations of *Drosophila melanogaster *[27], and other studies in laboratory *Drosophila *populations have also presented values considerably below our estimate (see [27] for a brief review). Our N_e_/N ratios were also higher than the average values of 0.11 reported for natural populations [28]. These higher values might be a result of the lower fluctuations in the overall census size of our laboratory populations through time, compared to other laboratory studies or to what is expected to occur in wild populations. However, given the abrupt transformation imposed on the demographic structure on our populations as a result of their recent sampling from the wild, this cross-study comparison has to be made with caution.

### Testing for positive selection at the molecular level

It has been extensively documented that both AR and TW populations have undergone adaptation to laboratory conditions with respect to life-history traits, some of which show clear directional trends of improvement throughout laboratory culture [8]. For the microsatellite data, we obtained significant deviations from neutral expectations at locus *dsub14 *for both groups of AR and TW populations after 14 generations of laboratory adaptation. This was due to an increase in frequency of a low frequency allele in all 6 populations studied between generations 3 and 14 for that particular locus, suggesting that positive selection could have occurred in the region of this microsatellite. However, a significant increase in heterozygosity through time was observed at this locus and not a decline, which is the common expectation of a selective sweep (e.g. [12]). This increase in heterozygosity could be a transient effect on a locus with a low number of alleles and low heterozygosity, leading to higher heterozygosity resulting from a rise in the frequency of an initially rare allele [see Additional file 1]). The lower initial variability at this locus could in turn have been the result of selective constraints affecting this region in wild populations, although we cannot exclude low mutation rates as a possible explanation given the low number of repeats in this locus [29].

A strong point in favour of the action of directional selection near the *dsub14 *locus is that the allele showing an increased frequency was the same in all TW populations. However, this pattern is not ineluctable, since sampling effects in the formation of our replicate populations, particularly involving low frequency alleles, could have led to different linkage disequilibria between alleles at this microsatellite locus and positively selected alleles some distance away from *dsub14*. This may explain the pattern observed at this locus in the AR populations, where two alleles were involved, one common to the AR_1 _and AR_2 _populations, and a different one for AR_3_.

Between generations 14 and 40, microsatellite locus *dsub14 *showed a significant deviation from neutrality in AR but not in TW populations. During this period, the TW_2 _population underwent a drop in the frequency of the putative hitchhiking allele, leading to a significant decline in heterozygosity over this period. Moreover, the deviation from neutrality of the AR_3 _population was not due to changes of frequency in the expected direction, since there was a drop in frequency of the putatively selected allele. These results complicate the interpretation of our findings, since hitchhiking within a region undergoing directional selection is expected to lead to a consistent increase in the frequency of the hitchhiking allele and ultimately to its fixation, unless linkage is broken by recombination.

The continued monitoring of allele frequency change at this locus over subsequent generations could help to clarify the evolutionary forces acting on it. Also, since the number of loci involved in our screen of molecular variants is low, the analysis of other microsatellite loci adjacent to this particular locus, searching for signs of low polymorphism in the genomic region, may rule out the possibility of false positive results (see [30]). At the same time, sequence analysis of flanking regions could be useful in the search for candidate genes underlying phenotypic adaptation. In fact, its location in chromosome O could account for the hitchhiking effect involving *dsub14*, since this chromosome harbours considerable inversion polymorphisms in *Drosophila subobscura*, which limit recombination [31,32].

## Conclusion

We observed a depletion of genetic variability and an increase in genetic differentiation among our laboratory populations through time. This is the predicted outcome of genetic drift effects in populations with smaller sizes, relative to those that are characteristic in the natural environment. Different genetic backgrounds appear to have had limited impact on these drift effects, since laboratory populations founded from different wild sources did not differ in their rate of variability decline through time. Our data suggest that selection acting on life history traits interacts with genetic drift, particularly through the smaller effective population sizes at early stages of adaptation, leading to a steeper initial drop in molecular genetic variability. Finally, we also found evidence of positive selection at one of the ten molecular markers analyzed, although this inference should only be considered provisional at this point.

## Methods

### Foundation and maintenance of the laboratory populations

This study involves two synchronous laboratory foundations carried out in the autumn of 2001, one from Sintra, Portugal, called "TW", and another one from Arrábida, Portugal, called "AR" (the two localities being 50 Km apart). The TW population was founded from 110 females and 44 males and the AR population began with 59 females and 24 males. After two generations in the laboratory, each population was split into three replicate populations, TW_1–3 _and AR_1–3_. From the moment of foundation, all populations were maintained under the same conditions: discrete generations of 28 days, reproduction close to peak fecundity, a controlled temperature of 18°C, and controlled densities (see [8,33]). Population sizes were usually between 600 and 1200 individuals.

### Microsatellite genotyping methods

AR and TW populations were genotyped for 10 microsatellite loci at generations 3, 14, and 40 after laboratory foundation. At each generation, 30 females were analyzed for each of the six populations studied (TW_1–3 _and AR_1–3_).

The ten microsatellite loci analyzed in this study were: *dsub01*, *dsub02*, *dsub05*, *dsub10*, *dsub14*, *dsub19*, *dsub20*, *dsub21*, *dsub23 *and *dsub27*. These markers had been previously identified and characterized in *D. subobscura *[29]. Loci *dsub05*, *dsub19 *and *dsub21 *are X-linked and the others are autosomal.

DNA for the microsatellite analysis was extracted from single flies using an extraction protocol described in [34]. PCR reactions were performed for a total volume of 25 μl with 2.5 pmol of each primer (10 μM), 3 μl dNTP's (1 mM), 2 μl 10 × buffer, 1 U *Taq *polymerase and 1 μl of DNA. All 10 loci were amplified using four different multiplex PCR reactions *(dsub02+dsub05; dsub10+dsub14; dsub20+dsub21+dsub27; dsub01+dsub19+dsub23)*. All reactions were performed on an ABI GeneAmp PCR System 2700 machine using the following steps: 5 min at 95°C, then 30 cycles of 1 min at 95°C, 1 min at 54°C and 30 s at 72°C followed by 5 min at 72°C. After amplification, the products were visualized in an agarose gel and then loaded on an ABI PRISM 310 sequencer (Applied Biosystems). Allele sizes were estimated by comparison to an internal size standard (GeneScan-500 ROX) using the software program Genotyper (Applied Biosystems).

### Statistical methods

#### Microsatellite analysis

##### a) Measures of genetic diversity and differentiation

Genetic variability was measured using both mean number of alleles per locus and mean expected heterozygosity with GENEPOP, version 3.2 [35].

Differences in genetic variability between AR and TW groups of populations in each generation were assessed using a bifactorial mixed ANOVA defining *group *(with two categories: AR and TW) as a fixed factor and *locus *as a random factor, with each genetic variability measure as a dependent variable. To test for differences in genetic variability in each group across generations, we applied a similar model, with *generation *as a fixed factor (with three categories: generations 3, 14, and 40) and *locus *as a random factor. The changes in microsatellite variability through time were studied by defining two *periods*: the first period between generations 3 and 14 and the subsequent period between generations 14 and 40. Rates of variability decline were calculated for each period for both AR and TW populations, using both allele number and expected heterozygosity (standardized by the square root of the number of generations of each period). Differences in the rates of variability decline between *periods *and *groups *were tested with trifactorial mixed ANOVAs (sigma-restricted, type III SS model) with *group*, *period *(fixed) and *locus *(random) as factors.

All parameters tested by ANOVA had a normal distribution of residuals. Rates of heterozygosity decline were arcsine transformed to meet ANOVA assumptions. All ANOVAs were performed using Statistica 5.0.

Genetic differentiation was accessed through a hierarchical design with the following levels: groups (or generations); populations within groups (or generations) and individuals within populations. All measures were calculated according to Wright's *F *statistics using the GDA software version 1.1 ([36]; see also [37]). These parameters included calculations at the following levels of hierarchy: at the group level (*F*_st groups _and *F*_st generations _described as θ_P _in the GDA software) and at the population level (*F*_st _described as θ_S _in the GDA software). The significance of the *F *statistics was evaluated using 95% confidence intervals (CIs) that were calculated by 1,000 bootstrap replicates of the loci.

Specifically, the following comparisons were performed in each generation analyzed (generations 3, 14 and 40): (a) Between the two groups of populations (*F*_st groups _; AR *vs*. TW); (b) Between populations from the two different groups (*F*_st_; AR populations *vs*. TW populations); (c) Between populations within each group (*F*_st_; i.e. differentiation between replicate populations from the same group, e.g. AR_1–3_). Comparisons between generations for each group (*F*_st generations_; e.g. TW at generation 3 *vs*. TW at generation 14; etc...) were also performed.

##### b) Estimating effective population sizes

Effective population sizes (N_e_) for each AR and TW population during laboratory adaptation were estimated from temporal microsatellite data using a pseudo-likelihood approach [26] and also through the loss of heterozygosity formula H_t_/H_0 _= (1-1/2N_e_)^t ^(see [38]). Likelihood-based methods were used because they provide more reliable N_e _estimates relative to classical methods (e.g., [39,40]), particularly for samples with many rare alleles [41,42]. The temporal method allows to estimate the effective population size through the analysis of the variation in the allele frequencies of temporally spaced samples of a given population [41]. This method calculates the standardized variance in the temporal changes of allele frequency *F*, which is reciprocally proportional to the effective population size. In our study, the N_e _estimates will thus represent the effective size of our populations during laboratory evolution and not that of the natural populations from which they have been derived.

Effective population sizes were estimated for the two periods (between generations 3 -14 and between generations 14 – 40), and also for the overall data (generations 3 to 40) in both AR and TW populations.

The pseudo-likelihood N_e _estimates were obtained using the MLNE program [26,42], given our temporally spaced samples for each AR and TW population. All analyses were performed allowing a maximum N_e _value of 1000.

##### c) Testing for positive selection

Effects of positive selection were tested for each microsatellite locus by applying the Ln RH test statistic [12]. This test is based on the comparison of the logarithm of the ratio between expected heterozygosities obtained for each locus in two populations: Ln RH = Ln [((1/(1- H_pop1_))^2^-1)/((1/(1- H_pop2_))^2 ^-1)]. The aim of this test is to search for loci with a pattern of variability which is significantly different from that expected with neutrality.

To apply this test, ratios of expected heterozygosities were calculated for each locus using data from generations 3 and 14 (G14/G3 ratios) and also generations 14 and 40 (G40/G14 ratios) for each AR and TW populations. To account for the different effective population sizes of X chromosomes, a correction was introduced for the X chromosomal loci heterozygosities (see [43]):

H_corr _= 1-1/[√1+*k*(1/(1-H_obs_)^2^-1)],

the correction factor *k *used was 1.33, assuming a balanced sex ratio [44]. Since Ln RH values are expected to follow a Z distribution for neutrally evolving microsatellite loci [11], significant deviations of standardized Ln RH values from this distribution indicate a putative selective sweep [12]. This test was applied for each AR and TW replicate population.

To detect potentially selected loci, we also performed a one-way ANOVA, defining *locus *as factor and the Ln RH values (of the three AR or TW populations) as the dependent variable. To search for differences between loci a *post hoc *Scheffé test was also performed. Normality in Ln RH data was previously tested. All these analyses were done in Statistica 5.0.

## Authors' contributions

PS, JS and MM performed the life history trait assays and maintained laboratory populations. PS and JS performed the microsatellite analyses. PS and MM carried out the statistical analyses. PS, MP, MRR and MM designed the experiment. PS and MM wrote the first draft of the manuscript. MP, MRR, and JS contributed to the final draft of the manuscript. All authors read and approved the final manuscript.

## Supplementary Material

Additional file 1 **Genetic Variability of AR and TW populations**. Mean allele number and expected heterozygosity per locus for the AR and TW groups of populations at generations 3, 14 and 40 of laboratory evolution. Click here for file

Additional file 2 **Pairwise *F*_*st *_comparisons between AR and TW populations**. Genetic differentiation between AR and TW populations at each of the three generations analyzed: generation 3, 14 and 40. Click here for file

Additional file 3 **Pairwise *F*_*st *_comparisons within and across laboratory generations**. Genetic differentiation in each set of replicate populations (AR or TW) within and across generations analyzed. Click here for file

## References

[B1] Futuyma DJ Evolutionary Biology 1998 3 Sunderland, MA: Sinauer Associates

[B2] Teotónio H Rose MR Variation in the reversibility of evolution Nature 2000 408 463 466 10.1038/35044070 11100726

[B3] Hartl DL Clark AG Principles of Population Genetics 1989 Sunderland, MA: Sinauer Associates

[B4] Cohan FM Can uniform selection retard random genetic divergence between isolated conspecific populations? Evolution 1984 38 495 504 10.2307/2408699 28555989

[B5] Cohan FM Hoffmann AA Uniform selection as a diversifying force in evolution: Evidence from *Drosophila* Am Nat 1989 134 613 637 10.1086/285000

[B6] Falconer DS Mackay TFC Introduction to Quantitative Genetics 1996 Harlow: Addison Wesley Longman

[B7] Rose MR Nusbaum TJ Chippindale AK Rose MR, Lauder GV Laboratory evolution: the experimental Wonderland and the Cheshire Cat syndrome Adaptation 1996 San Diego, CA: Academic Press 221 241

[B8] Simões P Rose MR Duarte A Gonçalves R Matos M Evolutionary domestication in *Drosophila subobscura* J Evol Biol 2007 20 758 766 10.1111/j.1420-9101.2006.01244.x 17305841

[B9] Morgan TJ Garland T Jr Irwin BL Swallow JG Carter PA The mode of evolution of molecular markers in populations of house mice under artificial selection for locomotor behavior Heredity 2003 94 236 242 10.1093/jhered/esg021 12816964

[B10] Rodriguez-Ramilo ST Moran P Caballero A Relaxation of selection with equalization of parental contributions in conservation programs: An experimental test with *Drosophila melanogaster* Genetics 2006 172 1043 1054 10.1534/genetics.105.051003 16299385 PMC1456204

[B11] Schlötterer C A microsatellite-based multilocus screen for the identification of local selective sweeps Genetics 2002 160 753 763 11861576 10.1093/genetics/160.2.753 PMC1461965

[B12] Kauer MO Dieringer D Schlötterer C A microsatellite variability screen for positive selection associated with the "Out of Africa" habitat expansion of *Drosophila melanogaster* Genetics 2003 165 1137 1148 14668371 10.1093/genetics/165.3.1137 PMC1462820

[B13] Harr B Kauer M Schlötterer C Hitchhiking mapping: A population-based fine-mapping strategy for adaptive mutations in *Drosophila melanogaster* Proc Natl Acad Sci USA 2002 99 12949 12954 10.1073/pnas.202336899 12351680 PMC130566

[B14] Goldstein DB Schlötterer C (Eds) Microsatellites: evolution and applications 1999 Oxford: Oxford University Press

[B15] Maynard Smith J Haigh J The hitch-hiking effect of a favourable gene Genet Res 1974 23 23 35 4407212

[B16] Kaplan NL Hudson RR Langley CH The "hitchhiking effect" revisited Genetics 1989 123 887 899 2612899 10.1093/genetics/123.4.887 PMC1203897

[B17] Slatkin M Hitchhiking and associative overdominance at a microsatellite locus Mol Biol Evol 1995 12 473 480 7739389 10.1093/oxfordjournals.molbev.a040222

[B18] Pascual M Aquadro CF Soto V Serra L Microsatellite variation in colonizing and paleartic populations of *Drosophila subobscura* Mol Biol Evol 2001 18 731 740 11319257 10.1093/oxfordjournals.molbev.a003855

[B19] Nei M Maruyama M Chakraborty R The bottleneck effect and genetic variability in populations Evolution 1975 29 1 10 10.2307/2407137 28563291

[B20] Allendorf FW Genetic drift and the loss of alleles versus heterozygosity Zoo Biology 1986 5 181 190 10.1002/zoo.1430050212

[B21] Hedrick PW Highly variable loci and their interpretation in evolution and conservation Evolution 1999 53 313 318 10.2307/2640768 28565409

[B22] Crandall KA Bininda-Edmonds ORP Mace GM Wayne RK Considering evolutionary processes in conservation biology: returning to the original meaning of "evolutionary significant units" Trends Ecol Evol 2000 15 290 295 10.1016/S0169-5347(00)01876-0 10856956

[B23] Reed DH Frankham R How closely correlated are molecular and quantitative measures of genetic variation? A meta-analysis Evolution 2001 55 1095 1103 11475045 10.1111/j.0014-3820.2001.tb00629.x

[B24] Reed DH Frankham R Correlation between fitness and genetic diversity Conserv Biol 2003 17 230 237 10.1046/j.1523-1739.2003.01236.x

[B25] Berthier P Beaumont MA Cornuet JM Luikart G Likelihood-based estimation of the effective population size using temporal changes in allele frequencies: a genealogical approach Genetics 2002 160 741 751 11861575 10.1093/genetics/160.2.741 PMC1461962

[B26] Wang J A pseudo-likelihood method for estimating effective population size from temporally spaced samples Genet Res Camb 2001 78 243 257 10.1017/s0016672301005286 11865714

[B27] Briscoe DA Malpica JM Robertson A Smith GJ Frankham R Banks RG Barker JSF Rapid loss of genetic variation in large captive populations of *Drosophila *flies: implications for the genetic management of captive populations Conserv Biol 1992 6 416 425 10.1046/j.1523-1739.1992.06030416.x

[B28] Frankham R Effective population size/adult population size ratios in wildlife: a review Genet Res Camb 1995 66 95 107 10.1017/S0016672308009695 18976539

[B29] Pascual M Schug MD Aquadro CF High density of long dinucleotide microsatellites in *Drosophila subobscura* Mol Biol Evol 2000 17 1259 1267 10908646 10.1093/oxfordjournals.molbev.a026409

[B30] Wiehe T Nolte V Zivkovic D Schlotterer C Identification of Selective Sweeps Using a Dynamically Adjusted Number of Linked Microsatellites Genetics 2007 175 207 218 10.1534/genetics.106.063677 17057237 PMC1775015

[B31] Hoffmann AA Sgrò CM Weeks AR Chromosomal inversion polymorphisms and adaptation Trends Ecol Evol 2004 19 482 488 10.1016/j.tree.2004.06.013 16701311

[B32] Munté A Rozas J Aguadé M Segarra C Chromossomal inversion polymorphism leads to extensive genetic structure: a multilocus survey in *Drosophila subobscura* Genetics 2005 179 1573 1581 15687280 10.1534/genetics.104.032748 PMC1449531

[B33] Matos M Rose MR Rocha Pité MT Rego C Avelar T Adaptation to the laboratory environment in *Drosophila subobscura* J Evol Biol 2000 13 9 19 10.1046/j.1420-9101.2000.00116.x

[B34] Gloor GB Preston CR Johnson-Schlitz DM Nassif NA Phillis RW Benz WK Robertson HM Engels WR Type I repressors of P element mobility Genetics 1993 135 81 95 8224830 10.1093/genetics/135.1.81 PMC1205629

[B35] Raymond M Rousset F GENEPOP (version 1.2): Population genetics software for exact tests and ecumenicism J Heredity 1995 86 248 249

[B36] Lewis PO Zaykin D Genetic Data Analysis: Computer Program for the Analysis of Allelic Data, Version 1.1; 2001 Free program available at http://www.eeb.uconn.edu/people/plewis/software.php

[B37] Weir BS Genetic Data Analysis II 1996 Sunderland, MA: Sinauer Associates

[B38] Crow JF Kimura M An Introduction to Population Genetics Theory 1970 New York: Harper & Row

[B39] Nei M Tajima F Genetic drift and estimation of effective population size Genetics 1981 98 625 640 17249104 10.1093/genetics/98.3.625 PMC1214463

[B40] Waples RS A generalized approach for estimating effective population size from temporal changes in allele frequency Genetics 1989 121 379 391 2731727 10.1093/genetics/121.2.379 PMC1203625

[B41] Wang J Estimation of effective population sizes from data on genetic markers Phil Trans R Soc B 2005 360 1395 1409 10.1098/rstb.2005.1682 16048783 PMC1847562

[B42] Wang J Whitlock MC Estimating effective population size and migration rates from genetic samples over space and time Genetics 2003 163 429 446 12586728 10.1093/genetics/163.1.429 PMC1462406

[B43] Kauer MO Zangerl B Dieringer D Schlötterer C Chromossomal patterns of microsatellite variability contrast sharply in African and Non-African populations of *Drosophila melanogaster* Genetics 2002 160 247 256 11805060 10.1093/genetics/160.1.247 PMC1461951

[B44] Pascual M Mestres F Serra L Sex-ratio in natural and experimental populations of *Drosophila subobscura *from North America J Zool Syst Evol Research 2004 42 33 37 10.1046/j.0947-5745.2003.00237.x

