# Evolutionary dynamics of molecular markers during local adaptation: a case study in *Drosophila subobscura*

**DOI:** 10.1186/1471-2148-9-133

**Published:** 2009-06-12

**Authors:** Pedro Simões, Marta Pascual, Josiane Santos, Michael R Rose, Margarida Matos

**Affiliations:** 1 Universidade de Lisboa, Faculdade de Ciências da Universidade de Lisboa, Centro de Biologia Ambiental, Departamento de Biologia Animal, Campo Grande, 1749-016 Lisboa, Portugal; 2 Department of Genetics, Faculty of Biology, University of Barcelona, 08028 Barcelona, Spain; 3 Department of Ecology and Evolutionary Biology, University of California, Irvine, California 92697-2525, USA

## Abstract

Here we present a correction to our article "Evolutionary dynamics of molecular markers during local adaptation: a case study in *Drosophila subobscura*". We have recently detected an error concerning the application of the Ln RH formula – a test to detect positive selection – to our microsatellite data. Here we provide the corrected data and discuss its implications for our overall findings. The corrections presented here have produced some changes relative to our previous results, namely in a locus (*dsub14*) that presents indications of being affected by positive selection. In general, our populations present less consistent indications of positive selection for this particular locus in both periods studied – between generations 3 and 14 and between generation 14 and 40 of laboratory adaptation. Despite this, the main findings of our study regarding the possibility of positive selection acting on that particular microsatellite still hold. As previously concluded in our article, further studies should be performed on this specific microsatellite locus (and neighboring areas) to elucidate in greater detail the evolutionary forces acting on this specific region of the O chromosome of *Drosophila subobscura*.

## Correction

We have recently detected an error in our article [1], concerning the application of the Ln RH formula to our microsatellite data. This necessitates some changes in the results and figures presented in the "*Testing for positive selection during laboratory adaptation" *results section. Here we provide the corrected data and discuss its implications for our overall findings.

Corrected Ln RH values comparing generations 3 and 14 remain significantly different between loci in both TW and AR populations (one-way ANOVA; *p *< 0.001). Standardized Ln RH values for microsatellite locus *dsub14 *fall outside the 95% confidence interval of the standard normal distribution for AR_1 _(*p *< 0.03). AR_2 _and AR_3 _populations show a marginally significant deviation from expectation of neutrality (*p *< 0.06 for AR_2_; *p *< 0.07 for AR_3_). But AR populations present less consistent indications of positive selection between generations 3 and 14 than previously indicated [1]. In addition, standardized Ln RH values for *dsub14 *between generations 3 and 14 in TW populations no longer differ significantly from neutral expectation, despite the fact that the Ln RH values for these populations remain high (Fig. 1).

**Figure 1:**
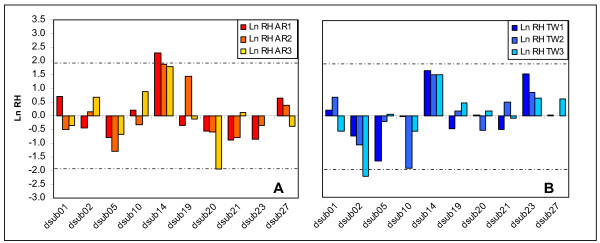
**Standardized Heterozygosity ratios (Ln RH) between generations 3 and 14**. Ln RH ratios (H14/H3) for AR (Fig. 1A) and TW (Fig. 1B) populations. Dashed lines represent the 95% confidence interval of the standardized normal distribution.

Between generations 14 and 40, corrected Ln RH values are not significantly different across loci either for TW or AR populations (one-way ANOVA; *p *> 0.05), though they were significant in our previous analysis for the AR data. Standardized Ln RH values for *dsub14 *fall outside the 95% confidence interval for AR_2 _(*p *< 0.04) and outside the 90% marginal confidence interval for AR_1 _(*p *< 0.06) – see Fig. 2, in contrast with the significant deviations previously reported for all AR populations [1].

**Figure 2:**
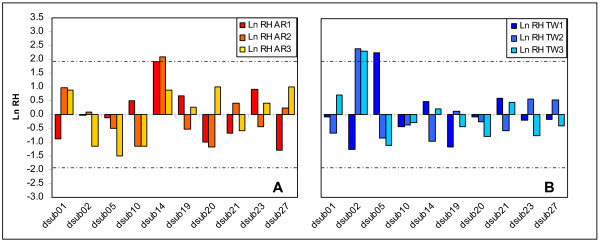
**Standardized Heterozygosity ratios (Ln RH) between generations 14 and 40**. Ln RH ratios (H40/H14) for AR (Fig. 2A) and TW (Fig. 2B) populations. Dashed lines represent the 95% confidence interval of the standardized normal distribution.

A new analysis that includes the wider range of generations analyzed (40 versus 3, Fig. 3) indicates a significant deviation pattern for locus *dsub14 *in all AR populations (*p *< 0.02 for AR_1_; *p *< 0.03 for AR_2 _and AR_3_), the TW_3 _population (*p *< 0.03), and a marginally significant deviation for TW_1 _(*p *< 0.07). Furthermore, as we already stated in our paper, the high Ln RH values in locus *dsub14 *were caused by the increase of an initially low-frequency allele in all populations analyzed, which is an observation in favour of positive selection acting near this marker.

**Figure 3:**
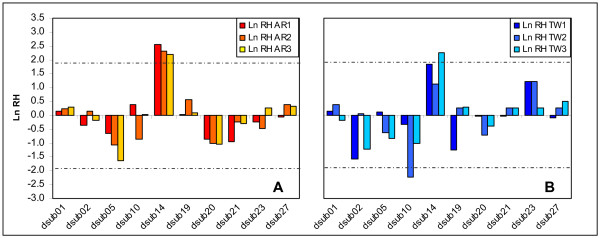
**Standardized Heterozygosity ratios (Ln RH) between generations 3 and 40**. Ln RH ratios (H40/H3) for AR (Fig. 3A) and TW (Fig. 3B) populations. Dashed lines represent the 95% confidence interval of the standardized normal distribution.

In general, we conclude that our main findings regarding the action of positive selection in our study hold, since our data is still suggestive of a deviation from neutral expectations in locus *dsub14*, although the signal is less pronounced than reported before. Nevertheless, as previously concluded [1], more studies should be conducted in this specific microsatellite locus (and neighboring areas) to further elucidate the evolutionary forces acting on this specific region of the O chromosome.

We regret any inconvenience that this error in our data might have caused the readers.
